# TERMS AND MEASURES OF COGNITIVE AGING AND COGNITIVE HEALTH: A SCOPING REVIEW

**DOI:** 10.1093/geroni/igz038.2213

**Published:** 2019-11-08

**Authors:** Christina E Miyawaki, Kelly Quinn, Raina Croff, Mia T Vogel, Basia Belza, Anita M Souza, Minhui Liu, Valerie J Edwards

**Affiliations:** 1 Graduate College of Social Work, University of Houston, Houston, Texas, United States; 2 University of Illinois at Chicago, Chicago, Illinois, United States; 3 Oregon Health & Science University, Portland, Oregon, United States; 4 Washington University in St. Louis, St. Louis, Missouri, United States; 5 University of Washington, Seattle, Washington, United States; 6 Johns Hopkins University, Baltimore, Maryland, United States; 7 Centers for Disease Control and Prevention, Atlanta, Georgia, United States

## Abstract

The Healthy Brain Initiative: National Public Health Road Map to Maintaining Cognitive Health (2007) called on the research community to more widely disseminate its work on cognitive aging and cognitive health. However, communication beyond individual disciplines is complex. We identified terminology that social scientists use to describe cognitive aging and cognitive health among older adults, demonstrated how such terms are defined, and illustrated how these constructs are being measured. We searched terms such as Alzheimer* and dementia in studies between 2007 and 2018 (n=209). Geriatrics (n=95), neurology (n=81), psychiatry (n=65), and psychology (n=30) were most common disciplines; however, there was no consistency in how terms were used within and across disciplines. A detailed review of “cognitive impairment” and “mild cognitive impairment” demonstrated that formal definitions were provided infrequently and measurement of constructs ranged widely. The variability in terminology, definitions and measures reflects a need for greater specificity in research communication.

